# Menopause mysteries: the exosome-inflammation connection

**DOI:** 10.1186/s13048-025-01591-9

**Published:** 2025-01-23

**Authors:** Aarushi Sultania, Subhashini Brahadeeswaran, Aparna Eledath Kolasseri, Sivaraman Jayanthi, Ramasamy Tamizhselvi

**Affiliations:** School of Biosciences and Technology, Vellore Institute of Technology, Tamil Nadu, Vellore, 632014 India

**Keywords:** Cell signaling, Follicular fluid exosomes, Inflammaging, Menopause, Ovarian aging

## Abstract

Extracellular vesicles, or exosomes, are produced by every type of cell and contain metabolites, proteins, lipids, and nucleic acids. Their role in health and disease is to influence different aspects of cell biology and to act as intermediaries between cells. Follicular fluid exosomes or extracellular vesicles (FF-EVs) secreted by ovarian granulosa cells are critical mediators of ovary growth and maturation. The movement and proteins of these exosomes are crucial in the regulation of cellular communication and the aging of cells, a process termed inflammaging. Menopause, a natural progression in the aging of females, is often accompanied by numerous negative symptoms and health issues. It can also act as a precursor to more severe health problems, including neurological, cardiovascular, and metabolic diseases, as well as gynecological cancers. Researchers have discovered pathways that reveal the diverse effects of exosome-driven cellular communication and oocyte development in the follicular fluid. It also explores the complex functions of FF exosomal proteins in the pathologies associated with menopause.

**Graphical Abstract:**

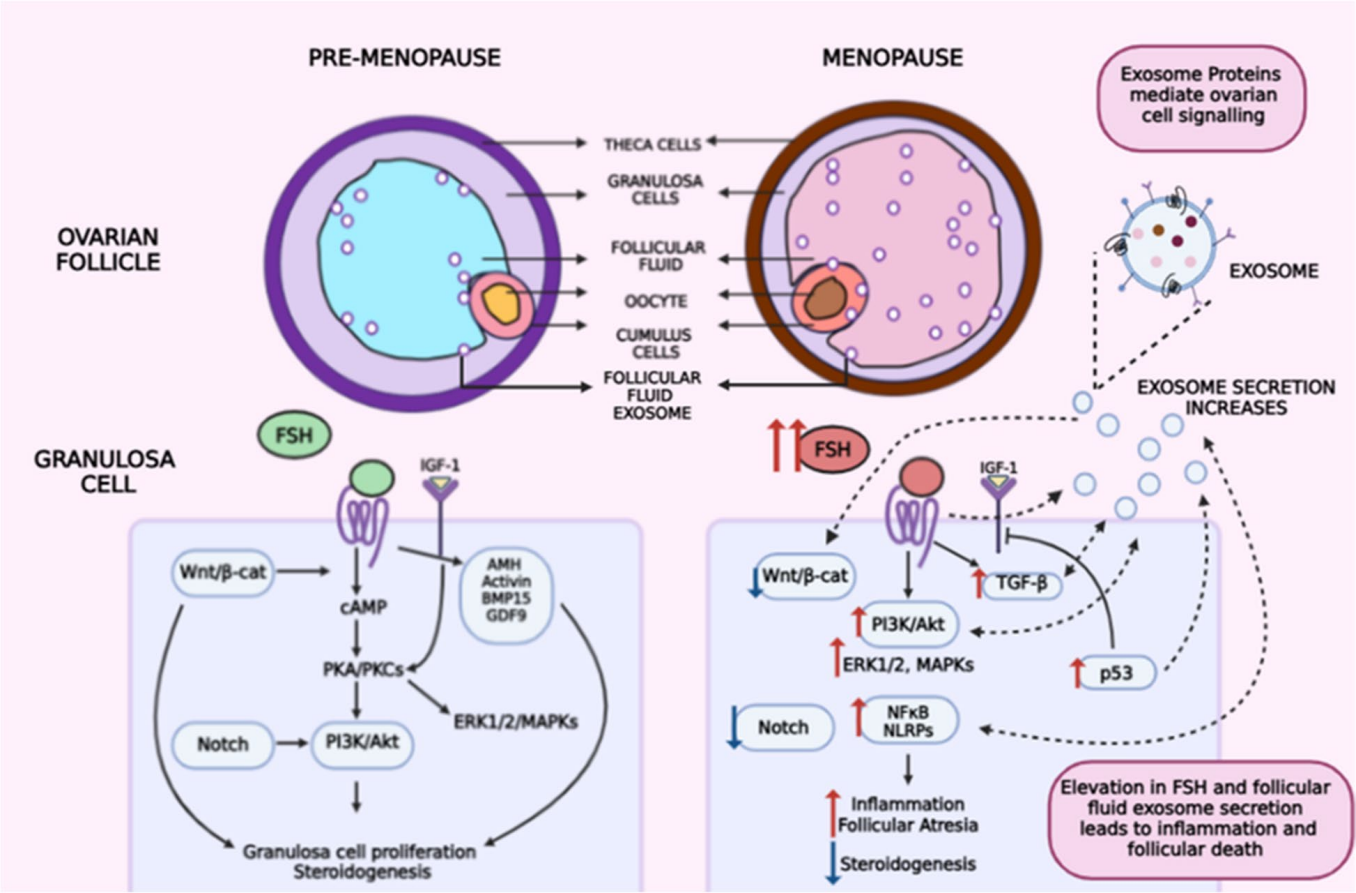

## Introduction

The female reproductive cycle is multistage; it commences with the onset of the menstrual cycle (menarche) and culminates in menopause [1]. During female fetal life, primary oocytes with their surrounding somatic or granulosa cells form “primary follicles”. During puberty, under the influence of gonadotropin hormones, such as follicle-stimulating hormone (FSH) and luteinizing hormone (LH), some ovarian follicles differentiate into secondary follicles and, subsequently preantral follicles. These hormones stimulate the synthesis of the female reproductive hormones - estrogen and progesterone, which further encourage folliculogenesis and secondary reproductive features. Finally, in response to a sudden elevation in LH (LH surge), mature oocytes are released from large preantral follicles [2, 3]. Follicle development is also influenced by non-gonadotropin anti-Mullerian hormone (AMH), which can be a reliable marker for diminishing ovarian reserve and ovarian aging [4]. Cell signaling and growth factors play equally important roles as catalysts of ovarian cell proliferation, apoptosis, and inflammation [5].

Menopause is the permanent loss of ovarian function and fertility, characterized by the irreversible cessation of the menstrual cycle in females. Usually, menopause is diagnosed after a complete year without menstrual bleeding or “amenorrhea” [1]. It is a natural aging process, with 45-56 years being the average age at diagnosis [6]. Estrogen and progesterone levels decline markedly during menopause, causing follicular loss or atresia. This increases significantly after the age of 35 [7]. As a regulatory mechanism, FSH secretion is increased. Therefore, menopausal females report abnormal serum hormonal levels of FSH (above 20 IU/mL) [8] and low estradiol or estrogen (below 20 pg/mL) [9]. With age, the ovarian microenvironment gradually transforms - pathways involved in cell proliferation and steroidogenesis, such as the Wnt and Notch pathways, may be subdued, whereas inhibitory pathways are dominant. Various studies have reported the upregulation of TGF-β, PI3K/Akt, and insulin signaling in aged females [5, 10–13]. These pathways contribute to reproductive senescence through their proapoptotic, proinflammatory, and antisteroidogenic effects. The expression of proinflammatory cytokines and pathways is also increased in the absence of estrogen-mediated protection. Increased oxidative stress and inflammation are key triggers for follicular degradation, DNA damage, and poor oocyte quality [14–16]. This manifests as an array of undesirable menopausal and perimenopausal symptoms such as vaginal atrophy, hyper fibrotic ovaries, loss of urinary control, hot flashes, mood disturbances, depression, and increased susceptibility to cardiovascular, neurological, and metabolic disorders, as well as cancers [17].

Granulosa cell-secreted extracellular vesicles or follicular fluid exosomes have been identified as critical mediators of ovarian cell signaling and inflammation. These subcellular nanosized packages are derived from the late endosome and are loaded with proteins, lipids, and RNAs (siRNAs, miRNAs, long noncoding RNAs, and mRNAs) that can be transmitted through the ovarian follicular fluid between somatic and germ cells, facilitating intracellular communication [18]. By allowing the exchange of various cell signaling and growth factors and through Wnt/β-catenin, PI3K/Akt, Notch, Hedgehog, and insulin signaling, and the MAPK/ERK inflammatory cascade, they stimulate cell proliferation, and steroidogenesis, as well as inflammation, apoptosis, and aging [5, 19]. Follicular fluid exosomes were first isolated from horse ovaries, with the choice of sources later expanding to humans, rats, mice, cows, and pigs [20]. They have also been implicated in ovarian afflictions such as polycystic ovarian disorder, primary ovarian insufficiency, and cancer [19, 21].

Ovarian aging-related experiments have revealed that modifications in follicular fluid (FF) exosome contents or cargo are responsible for follicular atresia and menopause. Specific FF exosome miRNAs related to cell signaling and apoptosis are downregulated or upregulated in aged females, leading to a permanent loss in fertility [10, 22]. The rate of follicular fluid exosome secretion is also increased with age [23]. This is linked to the dysregulation of cellular pathways, protein and antioxidant production, and aging-related inflammation in the ovary [23]. The diagnostic and therapeutic potential of FF-exosomal miRNAs in ovarian aging has been established; these miRNAs regulate granulosa cell proliferation, and Wnt, PI3K/Akt, Notch, insulin, and TGF-β signaling [10]. However, the domain of exosomal proteins remains relatively untapped. Exosomal proteins are irreplaceable components of exosomes, and play extensive roles in their biogenesis, cellular uptake, and function. Therefore, they can prove to be key, quantifiable indicators of various diseases. During menopause, they exert pleiotropic effects on ovarian granulosa cells, through various endocytic and signaling pathways. In recent studies related to ovarian dysfunction, their effects have been elucidated. This review focuses on understanding the roles of follicular fluid exosome trafficking and exosomal proteins in regulating cell signaling and inflammaging, which cause menopause-related pathologies.

## Follicular fluid exosomes: facilitators of ovarian function and aging

Extracellular vesicles (EVs) or exosomes are subcellular entities of 30-150 nm in diameter that originate in multivesicular bodies (MVBs), derived from late endosomes. The invagination of the MVB membrane leads to the formation of blebs or vesicles, which are loaded with RNAs, proteins, and lipids, and secreted from the cell. Secreted extracellular vesicles are endocytosed by recipient cells via clathrin-dependent or independent pathways [24, 25]. The exosomal cargo has multiple diverse effects on recipient cell growth, differentiation, and cellular processes. They have been implicated in several diseases, such as metabolic disorders such as obesity and diabetes, neurodegenerative disorders, cancers, and aging [26].Since exosome biogenesis and secretion are strongly influenced by an individual’s lifestyle choices and physiological state, exosomal cargo and number may be modified under diseased conditions. Overall, exosomes show great potential as both therapeutic agents, and diagnostic markers [27].

Follicular fluid extracellular vesicles (FF-EVs) have been isolated from the ovarian follicle fluid of many mammals. Typically, bovine, porcine, or equine follicular fluids are collected for research studies [20]. It is easier to isolate high amounts of FF-EVs from females undergoing in-vitro fertilization (IVF) treatments [28]. This is because the patients are administered FSH for hyperovulation. Fluid from pooled ovarian follicle samples is isolated via aspiration. Thereafter, for exosome isolation, differential centrifugation is carried out. The process can be sped up by size exclusion chromatography and commercial precipitation-based kits, such as ExoQuick. After enrichment, exosomes are characterized via transmission electron microscopy (TEM), scanning electron microscopy (SEM), dynamic light scattering (DLS), and nanoparticle tracking assay (NTA) [28, 29]. Human follicular fluid contains a diverse range of extracellular vesicles, varying in shape and size. The isolation of FF-EVs and microvesicles from females undergoing intracytoplasmic sperm injection treatments yielded 4 x 10^10^ particles/mL of follicular fluid, with a size range of 10-300 nm [28]. Exosomal RNA cargos are analyzed using real-time PCR or RNA sequencing, and protein profiles can be obtained via mass spectrometry [28].

FF-EVs and their constituent miRNAs and proteins facilitate ovarian growth and development by transmitting through ovarian gap junctions and transzonal projections [30]. Human FF-EVs are known to regulate oocyte maturation and follicular senescence through their constituent miRNAs(J. Zhang et al., 2019). A study in porcine granulosa cells demonstrated the role of FF-EV miRNAs in granulosa cell proliferation, maturation, and steroidogenesis through the Wnt/β-catenin, PI3K/Akt, and MAPK/ERK pathways [20]. Several in-vitro and in-vivo models have established the role of FF-EVs and diagnostic exosomal miRNAs in gynecological ailments such as polycystic ovarian syndrome (PCOS) and primary ovarian insufficiency (POI), ovarian cancer, endometriosis, and preeclampsia [31, 32].

FF-EVs play a pivotal role in menopause, by demonstrating clear age-related changes. A comparative study revealed that, compared with young, fertile individuals, aged, menopausal females presented altered FF exosomal miRNA profiles, and smaller and twice as many FF exosomes. This was potentially caused by increased oxidative stress, and inflammation. The upregulation of the proapoptotic marker p53 promotes exosome assembly and secretion. Other cellular mechanisms, such as protein processing in the endoplasmic reticulum are dysregulated [15, 23, 33]. Additionally, the upregulation and downregulation of FF-exosome miRNAs related to cell signaling pathways such as the PI3K/Akt, TGF-β, insulin, IGF, Wnt, and Notch lead to infertility and aging [10, 22]. FF-exosomes exert multiple effects on ovarian cells through their constituent proteins. Some classical proteins in exosomes include surface tetraspanins (CD-63, 81, 82, and 9), major histocompatibility complexes (MHC I and II), endosomal sorting complex proteins (ESCRTs), ALIX (ALG-interacting protein X), TSG-101 (tumor susceptibility gene-101), heparan sulfates, integrins, glycoproteins, exosome uptake proteins (caveolins, clathrins, actin, dynamin), and secretion proteins (SNAREs and Rab-GTPases). Furthermore, exosomes harbor cell signaling factors, proinflammatory cytokines, heat shock proteins (HSP70 and 90), G-proteins, cytoskeletal proteins, and metabolic enzymes. Stretches of lipids such as ceramides and flotlins also allow exosome uptake and secretion [34–36].

In bovine FF exosomes, nearly 322 proteins have been identified, including ribosomal and heat-shock proteins, proteins related to the endoplasmic reticulum, the complement cascade, PI3K/Akt signaling, and cellular metabolism [37]. Bovine FF-EVs also promote the expansion of cumulus granulosa cells around the oocyte by allowing the expression of TNF-α, prostaglandin-endoperoxide synthase 2 (*Ptgs2*), and pentraxin-related protein 3 (*Ptx3*) [38]. Comparative analysis between normal and PCOS patients revealed 3104 FF-EV proteins, of which 32 differentially expressed proteins were involved in metabolic processes such as cholesterol and ceramide synthesis, which are crucial for exosome biogenesis and uptake, and signal transduction. These studies suggest the critical role of FF exosomal proteins in ovarian function. With age, the metabolic pathways of the female ovary are altered; an increase in lipid and transfer RNA synthesis promotes the production of stress-related proteins in follicular fluid exosomes [29]. This review highlights several key stress-related FF-EV proteins involved in menopause-related changes.

## Beyond the change: understanding menopause-related afflictions

The transition to menopause is gradual and can take several years. Postmenopause, females are more prone to cardiovascular diseases, kidney dysfunction, osteoporosis, metabolic disorders such as diabetes mellitus, obesity, metabolic syndrome [21], and gynecological cancers. Although menopause is a natural phase in a female’s life, menopause-related symptoms and afflictions can make life adverse. Approximately 85% of females worldwide experience at least one undesired menopause-related symptom [39]. A recent Indian study reported that the quality of life for females postmenopause was poor because of mental health, sexual and urogenital problems, and increased susceptibility to diseases [40].

Genetic and lifestyle factors can accelerate ovarian senescence and infertility. Primary ovarian insufficiency (POI) or premature ovarian failure (POF) affects females below the age of 40. They present symptoms similar to those of menopausal and perimenopausal females and display menopausal hormonal patterns [41, 42]. POI often coincides with other disorders, such as diabetes mellitus, thyroid-related deficiencies, cardiovascular diseases, and autoimmune disorders [43]. Polycystic ovarian syndrome (PCOS), characterized by improper ovarian follicle growth, androgen excess, and anovulation, shares some common features with menopause, such as dysregulated granulosa cell signaling, and increased ovarian inflammation, apoptosis, and fibrosis. Menopause in the case of PCOS is starkly different from non-diseased conditions. It manifests as lower FSH levels and higher AMH levels, delaying the onset of menopause by 2 years. Impaired glucose and lipid metabolism increase the susceptibility of PCOS-menopausal individuals to cardiovascular diseases, metabolic disorders, depression and anxiety, and endometrial cancers. General symptoms of menopause, such as hot flashes, vaginal dryness, mood fluctuations, and low libido, are also more pronounced. This makes the menopausal transition and life postmenopause particularly difficult in PCOS-affected females [44–46]. With the increasing incidence of PCOS, understanding the unique PCOS-menopause pathophysiology becomes crucial. Nearly all females suffer from menopause-related issues, yet the amount of research carried out in this field is abysmally low. Thus, there is a need to define the physiological mechanisms behind adverse menopause symptoms.

## Menopause-related cell signaling and apoptotic pathways

The present general characteristics of important signaling pathways are outlined here to help elucidate the architecture of the key signaling network involved in menopause.

### Wnt/beta-catenin pathway

In the ovary, Wnt/β-catenin signaling is required for the proliferation of granulosa cells and maintenance of the ovarian reserve [47, 48] The canonical pathway involves Wnt ligands binding to their membrane receptors, Frizzled, and LRP5/6 (lipoprotein receptor protein), leading to the nuclear translocation of β-catenin. In the nucleus, β-catenin stimulates the transcription of cell proliferation genes [49, 50].

Knockout studies in mice and rats have proven the importance of Wnt4/Wnt2-related pathways in granulosa cell proliferation and steroidogenesis. The deletion of Wnt-4 impairs fertility in mice by reducing follicular size and number [47] On the other hand, the overexpression of the ligand leads to increased activation of β-catenin. This is crucial for steroidogenesis, as β-catenin enhances FSH/cAMP-mediated expression of the CYP19A1 (aromatase) gene for estradiol/estrogen production. The interaction of β-catenin with its downstream target, StAR (steroidogenic acute regulatory protein), is crucial for the synthesis of progesterone from cholesterol [47]. In Wntless (Wnt ligand transporters) null mice, fewer and smaller ovarian follicles, impaired luteinization, and low progesterone levels are observed [51].

Not surprisingly, Wnt ligands and their related signaling proteins are highly expressed in human ovarian tissue samples, with no age-associated decline [48]. Dysregulation of the Wnt pathway is a common feature of polycystic ovarian disorder (PCOS), and endometrial and ovarian cancer [52, 53] and can also be correlated with several menopausal symptoms. Given its role in epithelial tissue maintenance, Wnt/β-catenin signaling can curtail vaginal atrophy. Through its role in bone cell proliferation and development, it can prevent reduced bone mass conditions such as osteopenia, and osteoporosis in menopausal females [54]. Wnt 2 and 3 also have an antidepressant nature under stress conditions [55].

In menopausal women, increased follicular fluid exosome secretion is consistent with the suppression of ovarian Wnt signaling. Wnt signaling is regulated by ceramide-dependent exosome trafficking pathways. Membrane ceramides, which are increasingly produced in the inflamed ovary [37], allow for the sorting of tetraspanins into exosomes. Exosome tetraspanins CD9 and CD82 increase the exosomal export of β-catenin, depleting its nuclear and cellular levels, preventing cell proliferation and steroidogenesis [56]. Menopause-related hallmarks such as follicular senescence and low hormone production are consequences of decreased cellular β-catenin through increased exosome trafficking.

### TGF-β/SMAD signaling

The transforming growth factor-β (TGF-β) superfamily is a group of conserved cytokines that includes three human subtypes: TGF-βs (TGF-β-1, 2, 3), bone morphogenetic factors (BMPs), activin, nodal, anti-Mullerian hormone (AMH), and growth-differentiating factors (GDFs). This pathway is mediated by the phosphorylation of receptor-regulated SMADs or R-SMADs, which allow the transcription of various genes associated with cell growth and apoptosis [57]. TGF-β acts as a potent tumor suppressor in epithelial cells, inducing cell cycle arrest via p15, p21, p27, and p57 synthesis, and suppressing the proliferative gene c-myc [58]. It triggers reactive oxygen species formation, inhibits antioxidative enzymes, and activates inflammatory pathways [59, 60]. In endothelial and mesenchymal cells, TGF- β supports proliferation and angiogenesis [61].

**Fig. 1:**
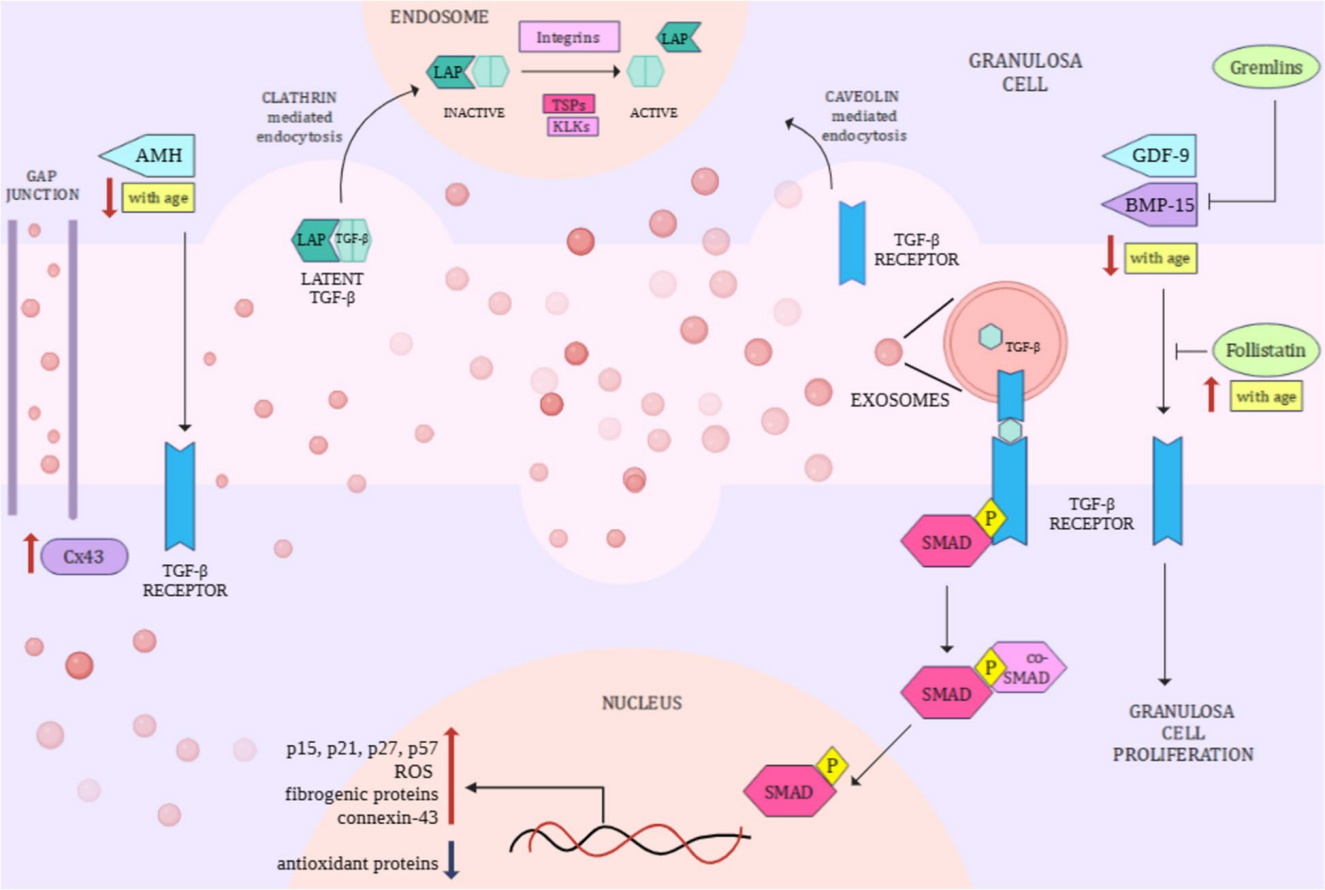
TGF-β signaling in granulosa cells – TGF-β signaling is exosome-mediated and facilitated by SMADs. The TGF-β ligand is activated in early endosomes in the presence of integrins, thrombospondins (TSPs), and kallikreins (KLKs), which are abundant in hyperfibrotic and aging ovaries. The TGF-β ligands GDF-9 and BMP-15 promote granulosa cell proliferation and are downregulated with age and inhibited by follistatin. Anti-Mullerian hormone (AMH) is also downregulated with age. Consequently, antioxidant stress, fibrogenesis, apoptosis, and exosome secretion increase

TGF-β signaling plays a dual role in ovarian function, by promoting reproductive senescence, and follicular development. While TGF-β signaling is usually linked to apoptosis, inflammation, and fibrosis in the ovary, BMP 2, 4, 15, GDF-9 ligands and AMH promote granulosa cell proliferation in an autocrine or paracrine manner [62]. GDF-9 also simultaneously activates inhibitory BMP antagonists (gremlins) to regulate ovarian cell growth. The proliferative effects of BMP-15 are countered by follistatin, a protein found in granulosa cells and follicular fluid [63, 64] (Fig. 1).

A gene expression study in mouse oocytes confirmed an increase in TGF-β in aged samples [65]. A *Caenorhabditis elegans* model for ovarian aging established those deleterious mutations in R-SMADs improved oocyte quality and fertility and reduced DNA damage, delaying reproductive senescence [66]. Estrogen, abundant in healthy ovaries, inhibits TGF-β signaling through the degradation of SMADs in vitro [67]. Interestingly, serum follistatin levels are elevated in postmenopausal women [68], while follicular fluid GDF-9 and BMP-15 levels decline [69]. Low AMH levels in postmenopausal women further reflect diminishing ovarian reserve [70].

TGF-β signaling can be further linked to other menopausal symptoms. Overexpression of TGF-β1 and TGF-β receptors in the postmenopausal endometrium inhibits epithelial cell differentiation and promotes atrophic conditions [71, 72]. The pathway drives the transcription of profibrotic genes, epithelial-to-mesenchymal transition and extracellular matrix deposition. These functions are exacerbated by ROS production and FSH [73–75].

TGF-β receptors are internalized and recycled via clathrin and caveolin-mediated endocytosis. Additionally, caveolin-clathrin fused vesicles also transport TGF-β into early endosomes [76]. During menopause, these endocytic pathways may be upregulated, as caveolin synthesis increases with FSH elevation and reduced estrogen and progesterone [77]. Exosome-mediated TGF-β trafficking is more potent than free TGF-β transport. Inactive TGF-β1 complexes with LAP (latency-associated protein) are internalized by exosomes by binding to exosomal heparan sulfate glycoproteins. Once in the acidic environment of early endosomes, TGF-β dissociates from exosomes and gets activated [78, 79]. Cellular deposition of LAP-TGF-β1 is promoted by fibronectins and fibrillins, while extracellular matrix proteins (thrombospondins, kallikreins, and integrins) induce LAP cleavage [79]. These proteins are abundant in the hyperfibrotic ovary, amplifying exosome-mediated TGF-β signaling during menopause. In follicular fluid exosomes from individuals with PCOS, kallikreins, thrombospondins, and fibrinogens are overexpressed [37].

Several miRNAs and long noncoding RNAs present in the follicular fluid and its exosomes regulate TGF-β upregulation in aging females. Additionally, TGF-β signaling and FSH secretion promote the formation of gap junctions in granulosa cells by upregulating the expression of connexin 43. This increases intraovarian communication via exosomes. Conversely, proliferative TGF-β ligands such as BMPs downregulate Cx43 [80]. Thus, the cross-talk between TGF-β and exosomes results in age-related changes in the ovarian microenvironment.

### Notch signaling

Notch signaling is a key pathway in cell proliferation, differentiation, invasion, adhesion, cell-to-cell interactions, and apoptosis. The transcription of genes related to these processes is initiated by the binding of Delta-Serrate-Lag 2 (DSL) ligands to Notch receptors (1-4 in mammalian cells), causing proteolysis of the intracellular domain of Notch receptors (NICD) [81]. In the ovary, Jagged-mediated Notch signaling drives granulosa cell proliferation via the transcription of Hes/Hey and c-Myc genes, promotes differentiation through Foxl2, and survival through activins. In pregranulosa cells, proliferative TGF-β ligands GDF9 and BMP15, support Notch-2 receptor expression, strengthening oocyte–granulosa cell interactions [82]. For estrogen synthesis and luteal cell survival, Notch signaling further induces the PI3K/Akt pathway [83]. Delta-4 signaling promotes angiogenesis, follicle growth, and progesterone synthesis [84].

Notch dysfunction is common in ovarian disorders, such as PCOS, ovarian cancer, and POF. In a POF-mouse model, growth hormone-mediated Notch-1 activation aided oocyte maturation, ovarian tissue repair, and estrogen synthesis [85]. Similarly, overexpressing lncHOTAIR in hamster ovary cells upregulated Notch-1, alleviating POF symptoms [86]. An RNA-microarray study in mouse ovaries revealed the downregulation of Notch ligands in aged and perimenopausal samples [87]. Estrogen receptor-mediated Notch signaling protects the vascular endothelium from TNF-α-induced inflammation and apoptosis [88] and ameliorates cardiorenal dysfunction [89]. In middle-aged female rats, downregulation of the Notch pathway caused chronic stress and depression [90].

Exosomes and novel extracellular vesicles, ARMMs (arrestin domain-containing protein 1-mediated microvesicles), have been identified as carriers of Notch ligands and receptors [91–93]. Like TGF-β, Notch activation via endocytic pathways involves gamma-secretase-mediated cleavage of NICD under acidic endosomal conditions [94]. While there is no clear understanding of the exosomal regulation of Notch signaling, Notch inhibitors can play a significant role in ovarian aging. Numb is an endosomal inhibitor of Notch, which promotes the ubiquitination and lysosomal degradation of Notch receptors and DSL ligands (95 ,96). Numb is highly expressed in ovarian tissues, and its downregulation leads to the over proliferation of granulosa cells in ovarian cancer and endometriosis [95]. Therefore, its role in reproductive senescence is worth exploring.

### PI3K/AKT pathway

The PI3K/Akt/mTOR signaling pathway is essential for cell survival and plays a pivotal role in ovarian functions such as primordial follicle development, oocyte meiosis, the cell cycle, and granulosa cell survival. Activation by Kit-L (stem cell factor) triggers PI3K to convert PIP-2 to PIP-3, leading to phosphorylation of Akt, mTOR, and FOXO3a. mTOR promotes ribosomal protein synthesis and the secretion of TGF-β ligands GDF-9 and BMP15, while FOXO3a phosphorylation inactivates apoptosis pathways mediated by TNF-α, BCl, and FasL. Akt allows cell cycle arrest in case of DNA damage, via the checkpoint regulator Chk1. PI3K/Akt signaling is activated by estrogen, insulin signaling, and insulin growth factor (IGF-1) [96, 97].

Recent studies have shown that the overexpression of Akt has negative ramifications on ovarian health. The inhibition of PTEN (a PI3K antagonist) and hyperactivation of Akt lead to the loss of primordial follicles and increased DNA damage, via exacerbated Ras/Raf/MAPK signaling. In POI, PTEN inhibition hinders follicular growth and generates ROS, whereas mTOR inhibitors improve fertility and follicle numbers [97]. The PI3K/Akt pathway has been associated with ovarian aging in several animal and human studies, where mutations in Akt and cell cycle checkpoints impair DNA damage repair (DDR) [98–100].

Exosomal proteins are major contributors to PI3K signaling. A study in PCOS-affected individuals revealed 32 differentially expressed follicular fluid exosome proteins, some of which are involved in the PI3K/Akt pathway [101]. In bovine FF exosomes, 13 proteins crucial for exosome uptake and PI3K/Akt signaling have been identified: collagen alpha-1 or COL6A1; heat shock proteins such as HSP90AA1, HSP90AB1, and HSP90B1; integrins such as ITGA2, ITGA6, ITGAV, ITGB1, and ITGB3; immunoglobulin heavy chain constant Mu protein or IGHM; Rho GTPase RAC1; vitronectin or VTN; and signal transducer YHAQW [37]. The expression of the gap junction connexin Cx43 is increased by the phosphorylation of Akt [96].

### Glucose metabolism and insulin signaling

Glucose is the main energy substrate for ovarian cell growth, and both hypo and hyperglycemia can cause ovarian dysfunction and infertility. In follicular cells, glucose is utilized mainly via the AMPK and hexosamine pathways. In response to nutritional stress (hypoglycemia), the AMPK pathway prevents estrogen and progesterone synthesis, FSH-mediated cell proliferation, and promotes apoptosis via Akt. It also stimulates the production of proinflammatory growth factors, such as TGF-β, TGF-α, and FGF-2 [102].

Recent studies suggest that while insulin is necessary for oocyte and granulosa cell proliferation and steroidogenesis, at high doses, it can stimulate hypoglycemic conditions detrimental to ovarian growth. Follicular development is arrested in hyperinsulinemic mice [12]. Hyperinsulinemia and estrogen deficiency activate Ras/Raf/ERK signaling, causing many menopausal women to develop insulin resistance, gain abdominal weight, and become susceptible to diabetes mellitus [12, 13].

**Fig. 2:**
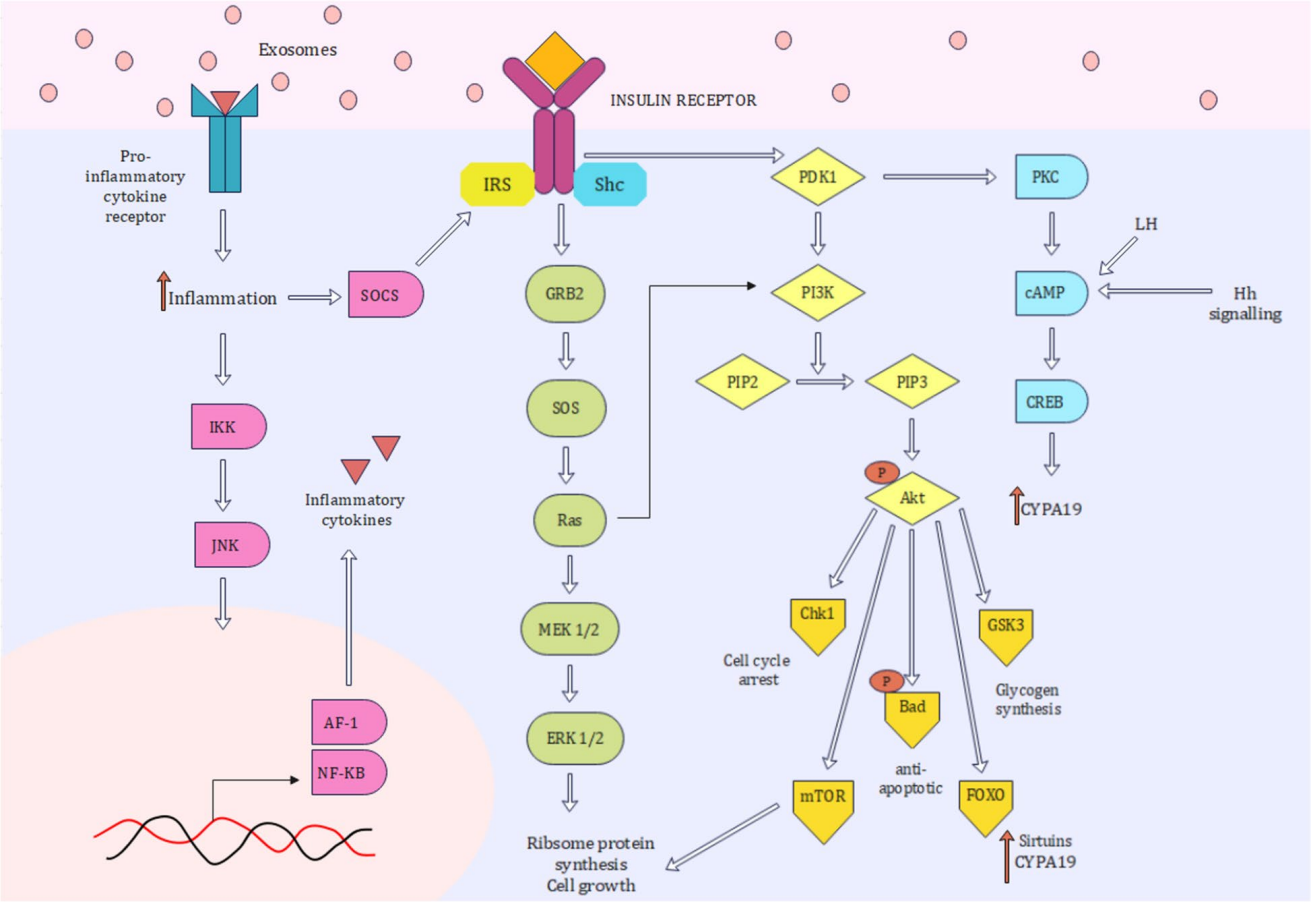
Insulin signaling promotes inflammatory Ras/MAPK signaling and PI3K/Akt signaling. Through the expression of Akt, it plays a role in monitoring oxidative stress, cell survival, cell cycle regulation, aromatase expression, ribosomal proteogenesis, and glycogen synthesis. Increased exosome secretion and inflammation encourage insulin signaling and may have negative effects on granulosa cell health

Insulin-like growth factor (IGF) signaling mirrors insulin signaling through the PI3K/Akt and MAPK/ERK pathways [103, 104]. It exerts antiapoptotic effects on ovarian cells and maintains oocyte quality [105]. It promotes the expression of GLUT1 (a glucose transporter) and thereby glucose uptake [106]. The ovarian secretion of IGF-1 is stimulated by somatotropin or growth hormone, which is downregulated with age. Many therapeutic interventions for POI and PCOS involve the administration of GH [107, 108]. In the postmenopausal atrophic endometrium, IGF-1 expression is suppressed [71]. Reduced serum levels of IGF-1 in aged females are coincident with decreased bone density and an increased risk of osteoporotic fractures [109, 110] (Fig. 2).

Follicular fluid exosomes mediate glucose uptake and insulin signaling. Exosomal genes related to IGF-1, MAPK, and PTEN are linked to diminished ovarian reserves. Porcine FF exosome miRNAs inhibit the tumour suppressor BTG2, promoting cell survival through insulin signaling and Akt [111]. In PCOS, elevated exosomal DENND1A.V2 protein promotes insulin and MAPK signaling, causing insulin resistance and infertility. DENND1A is a guanine nucleotide exchange factor for Rab35 and therefore facilitates exosome uptake [19]. Although it is implicated in PCOS, its role in menopause remains unexplored.

### Hedgehog pathway

In mammals, Hedgehog signaling (Hh) involves three ligands, the sonic hedgehog (Shh), desert hedgehog (Dhh), and Indian hedgehog (Ihh), which bind the transmembrane receptors Patched (PTCH) 1 and 2, enabling the activity of transmembrane Smoothened (SMO) and the transcription of Gli1,2, and 3 and related genes [112]. In the *Drosophila* ovary, Hedgehog signaling promotes granulosa cell proliferation [113]. It promotes follicular and oocyte development and maturation and progesterone synthesis in mammals [114–116]. In bovine ovarian follicles, aromatase gene (CYP19A1) expression increases with the expression of Ihh and PTCH1 [117].

CREB (cAMP response element binding protein) is a key proliferative gene that plays an important role in estrogen synthesis. Under the influence of IGF-1, FSH receptors induce high levels of cAMP, which allows CREB to activate the CRE (cAMP response element) promoter in the aromatase gene [118]. A *C. elegans* ovarian model revealed that downregulation of the TGF-β/Smad pathway decreases CREB expression, triggering a compensatory increase in Hedgehog ligand expression to maintain oocyte quality and delay reproductive senescence. With aging-related TGF-β upregulation, Hedgehog signaling decreases [119]. However, the exact mechanisms of Hedgehog signaling in aging are not fully understood. Although exosomes derived from various tissues carry Hedgehog ligands [120, 121], the effect of this pathway on exosome secretion is not understood.

### p53 pathway

P53 or TP53 (tumor protein 53) is a proapoptotic cellular marker that is often related to cancer progression. It is involved primarily in the regulation of the cell cycle at the G1/S checkpoint and DNA damage repair. It activates cell cycle inhibitors p21 and retinoblastoma, while inhibiting cell cyclins and cyclin-dependent kinases. p53 initiates apoptosis through Fas-L and the mitochondrial cell death cascade. In the aging ovary, p53 is activated in response to increased ROS production, inflammation, and DNA damage [122, 123]. Subsequently, it induces apoptotic and autophagic pathways and antagonizes IGF-1/PI3K/Akt/mTOR-mediated cell survival. p53 activates AMPK signaling to mitigate nutritional stress, hindering steroidogenesis and cell proliferation [124].

It is now understood that p53 is highly important for exosome secretion. It regulates the expression of endocytic proteins such as TSAP6 (tumor suppressor associated pathway 6), Chmp4C (charged multivesicular body protein 4C), and Caveolin-1 [124]. Caveolin-1 is crucial to the makeup of caveolae, which mediate the exosomal uptake of various growth factors, such as Notch, TGF-β, IGF-beta, and EGFR (epidermal growth factor protein) [77]. Chmp4C is a component of the ESCRT-3 complex and is important for protein sorting, MVB biogenesis, and vesicle formation. TSAP-6, a channel protein present on the endosomal, vesicular and plasma membranes, facilitates exosome secretion in response to DNA damage and oxidative stress. Deletion of TSAP-6 impairs exosome secretion in mice [125]. In menopausal females, several miRNAs related to the p53 pathway are differentially expressed compared to normal controls [23]. Thus, the p53 pathway may be linked to the increased number of FF exosomes observed during menopause.

## Ovarian aging: a consequence of increased inflammation

Reproductive function is influenced by inflammation, with ovulation itself being an inflammatory process. The LH surge triggers follicular rupture and the release of proinflammatory cytokines from ovarian cells. This activates several signaling pathways, including PI3K/Akt, PKCs, PKAs, and MAPK/ERK, which promote steroidogenesis, prostaglandin synthesis, and increase ROS production for the degradation of the follicular wall [126].

**Fig. 3:**
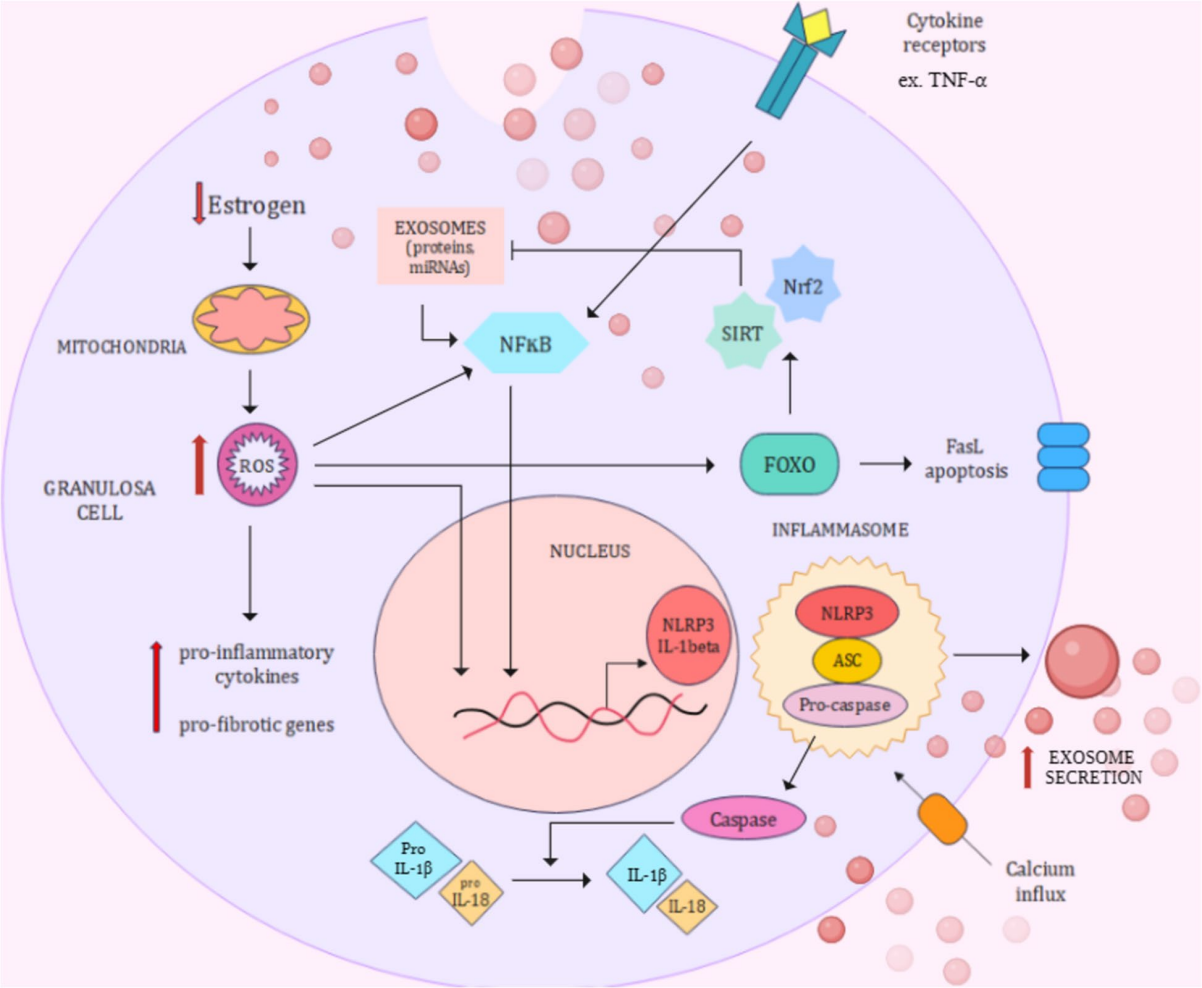
Exosome secretion and ovarian inflammation are interlinked; follicular fluid exosome cargo stimulates NF-κB/NLRP signaling and proinflammatory cytokines such as IL-18, IL-1β, and TNF-α. The NLRP inflammasome is ferried by exosomes, and thus, exosome secretion increases. Oxidative stress caused by a decrease in estrogen promotes inflammation and fibrosis. Antioxidants inhibit exosome secretion

However, chronic inflammation caused by a decrease in estrogen can accelerate aging. Before menopause, 17-β estradiol (estrogen) inhibits mitochondrial ROS formation by allowing the continuous expression of mitochondrial estrogen receptors [15, 127]. With age, the inflammatory cascade involving NF-κB, NLRP3 inflammasome, caspase-1, IL-1β, and TNF-α is increasingly activated [128]. ROS-related damage in ovarian tissue causes follicular atresia, ovarian cell apoptosis, impaired steroidogenesis, and abnormal angiogenesis. ROS trigger the production of proinflammatory cytokines, such as TNF-α, IL-1β, IL-1α, IL-6, IL-18, and IL-2, and chemokines, which are elevated in the serum and follicular fluid of aging females, as well as in the aging mouse ovary [14, 127, 129, 130] (Fig. 3).

Elevated oxidative stress is sensed by the FOXO protein, stimulating the release of antioxidants or Fas-L-mediated apoptosis. The antioxidant Sirtuin-1 activates FOXO and induces mitochondrial biogenesis. It is downregulated with reduced estrogen and AMH levels*.* In vitro knockdown of SIRT-1 and -2 leads to the formation of larger MVBs and increased exosome secretion [131]. The Nrf2 (nuclear factor E2-related factor) pathway, which decreases with age, is also an important defense against ROS (Mittal et al., 2014).

Extracellular vesicles sustain ovarian inflammaging—with declining estrogen levels, ovarian cells increasingly secrete EVs carrying NLRP inflammasomes [15]. Inflammasomes also stimulate EV secretion for the transmission of proinflammatory effectors of the NLRP/caspase-1 cascade, such as ILs. Calcium influx induces both inflammasome and exosome secretion [33]. In response to inflammation, exosomes polarize ovarian macrophages from the M1 to M2 phenotype, eliciting the secretion of profibrotic agents such as IL-3, IL-4, and TGF-β [132].

In PCOS, inflammatory follicular fluid exosomal proteins are increased many-fold. These include markers of oxidative stress and inflammation, such as S100A9, S100A8, peroredoxin (PRDX6, an antioxidant enzyme), C reactive protein (CRP), d‐DOPA‐ chrome decarboxylase (DDT), retinal dehydrogenase 1 (ALDH1A1), angiotensinogen, TNF-α, and kininogen‐1 (KNG1). When ovarian cells are treated with S100A9-enriched FF exosomes, the levels of TNF-α, IL-1, IL-6, IL-8, and chemokines are significantly increased, whereas the aromatase gene (CYP19A1) expression is suppressed. S100A9 and S100A8 activate the inflammatory cytokine cascade via NFκB [32].S100A8/A9 are upregulated in aged, multiparous mouse ovaries, and overexpressed in aging human ovarian tissues [133, 134].

A state of chronic ovarian inflammation is responsible for many menopause-related symptoms. Exosomes transport ovarian inflammasomes across various organs, contributing to cardiovascular and kidney diseases, metabolic disorders, skeletal disorders, and neuroinflammation [15]. Disruption of central nervous system activity leads to mood disorders and impairs memory and cognition [135]. NF-κB signaling activates bone-resorbing osteoclasts through receptor activator of NFκB (RANKL), causing skeletal disorders such as osteoporosis [136]. In response to NF-κB/NLRP-3 signaling, nitric oxide (NO) induces anti-inflammatory heat shock protein-70 (HSP-70) to prevent heat-related damage to cells. Heat flashes or vasomotor symptoms experienced during menopause are caused due to nitric oxide-mediated anti-inflammatory response. HSP-70 is a common exosome protein whose expression decreases with age and reduced Sirtuin levels [130]. Inflammation and oxidative stress associated with FF-exosome proteins can drive cancerous mutations and tumor growth, leading to gynecological cancers [137, 138].

Table 1 summarizes the key follicular fluid exosome proteins involved in menopause-associated cell signaling and inflammatory pathways, highlighting their potential roles in the regulation of cellular processes during this transition.

**Table 1:** Follicular fluid exosome proteins involved in menopause-related cell signaling and inflammation

Cellular function/pathway	Exosomal/Endosomal protein	Source	Mechanism of function	References
	TSAP6 (tumor suppressor associated pathway 6)	Endosome	Facilitates exosome secretion in response to DNA damage and oxidative stress.	[124, 125]
p53 pathway	Chmp4C (Charged multivesicular body protein 4C)	Endosome	Component of the ESCRT-3 complex. Aids MVB formation.	[124]
	Caveolin-1	Mammalian cell	Caveolin-mediated endocytosis of cell-signaling mediators like TGF-β, Notch, IGF, EGFR proteins.	[124]
Wnt signaling	Membrane tetraspanins CD9 and CD82	Exosome	Exosomal export of cellular β-catenin, downregulating Wntβ-catenin signaling	[56]
	TGF-β ligands	Exosome	Ferried by exosomes. Stimulate exosome secretion.	[139]
	Clathrin, SNARE proteins	Mammalian cell	Clathrin-mediated endocytosis of TGF-β containing exosomes.	[76]
	Caveolin	Mammalian cell	Caveolin mediated endocytosis of TGF-β exosomes.	[76]
TGF-β signaling	Heparan sulphate glycoproteins (membrane)	Exosome	Endosomal uptake of inactive TGF-β-LAP complex	[78]
	fibrinogens - fibronectins, fibrillins	Follicular fluid exosomes from PCOS affected females.	Cellular deposition of exosomal TGF-β-LAP complex. Promote fibrogenesis in the ovary.	[34]
	thrombospondins kallikreins integrins	Follicular fluid exosomes from PCOS-afflicted females.	Activation of TGF-β, by cleaving LAP. Promote hyperfibrotic and pro-inflammatory conditions in the ovary.	[34]
	connexin-43 (gap junction protein)	Granulosa cells	Form gap junctions between granulosa cells, and facilitate intercellular exosomal exchange. Upregulated by FSH and TGF-1. Downregulated by BMP-15 and GDF-9.	[63, 80]
Notch signaling	Notch ligands (Delta - DLLs, Serrate - Jagged 1 and 2, Lag) Notch receptors [1, 2]	Mammalian exosomes	Ferried by exosomes for sustained intercellular signaling.	[91, 92]
	Numb (Endosomal inhibitor of Notch)	Endosome	Promotes the trafficking of Notch ligands into late endosomes and lysosomes. Downregulated in ovarian cancers.	[140, 141]
PI3K/Akt signaling	COL6A1, HSP90AA1, HSP90AB1, HSP90B1, IGHM, ITGA2, ITGA6, ITGAV, ITGB1, ITGB3, RAC1, VTN, YWHAQ	Bovine follicular fluid exosomes	Proteins associated with exosome uptake. Upregulate PI3K/Akt signaling.	[37]
	Connexin-43 (gap junction protein)	Granulosa cells	Upregulated by Akt phosphorylation.	[96]
Insulin signaling	DENND1A.V2	Porcine follicular fluid exosomes	Guanine nucleotide exchange factor for Rab35. Facilitates clathrin-mediated endocytosis. Elevated in PCOS.	[19]
NLRP inflammasome cascade	NLRP protein complex or inflammasome	Ovary	Highly expressed in aged females. Stimulates the secretion of pro-inflammatory follicular fluid exosomes.	[142]
		Follicular fluid exosomes.	Carried and secreted by ovarian exosomes in response to decreased estrogen secretion.	[15]
Oxidative stress	Sirtuins (1 and 2)	Ovary	Antioxidant. Downregulated with age. Negatively correlated with exosome biogenesis – in vitro knockdown of SIRT-1 and 2 promotes exosome secretion.	[129, 131]
Inflammation	S100A9, S100A8	Follicular fluid exosomes	Pro-inflammatory, anti-steroidogenic FF exosomal proteins. Upregulated with age.	[32, 133, 134]
	peroredoxin (PRDX6, antioxidant enzyme) C reactive protein (CRP) d‐DOPA‐chrome decarboxylase (DDT) retinal dehydrogenase 1 (ALDH1A1) angiotensinogen TNF-α, kininogen‐1 (KNG1), S1009a	Follicular fluid exosomes	Pro-inflammatory exosomal proteins, activated in response to oxidative stress. Increased many-fold in FF exosomes of PCOS-affected individuals.	[32]
Heat shock response	HSP-70	Exosomes	Activated against heat stress by nitric oxide (NO) signaling. Associated with vasomotor symptoms in menopause. Anti-inflammatory. Downregulated with age.	[130]

## Conclusion

Follicular fluid exosomes are key elicitors of ovarian cell signaling. Since exosomes serve as a means of protein secretion from cells, they are rich in protein mediators that can initiate signal transduction in recipient cells. Exosomal proteins are protected against cellular degradation and are therefore abundant. In this review, the multifaceted roles of follicular fluid exosomal proteins in ovarian senescence and inflammation are discussed. During menopause, follicular fluid exosome secretion increases significantly in response to increased inflammation and oxidative stress. This is due to the activation of TGF-β and p53, as well as PI3K/Akt and insulin signaling. Pathways that promote granulosa cell proliferation and steroidogenesis, such as Wnt/β-catenin and Notch signaling, decline. A survey of various in vivo and in vitro studies has helped identify specific FF exosome proteins associated with these particular pathways. Exosomes are also correlated with adverse symptoms of menopause.

### Exosomes are promising therapeutic agents

While hormone therapy remains the most common intervention for menopause, its long-term side effects are a concern [143]. Alternatives such as metformin [144], an antidiabetic and anti-inflammatory drug, and resveratrol, an antioxidant [145], show potential but can have adverse effects at high doses [146]. Using exosomes as drug delivery vehicles offers a low-dose and targeted approach [147]. While current ovarian regeneration therapies involve mesenchymal stem cell exosomes [148, 149], in the future, FF exosome-based therapies may be devised. Just as the administration of EVs from young plasma mitigates aging-related dysfunctions [150], EVs from young follicular fluid may help reverse menopause-related adverse effects. In one study, a follicular fluid exosomal miRNA mitigated aging-related oxidative stress [151]. Such interventions can be especially beneficial for perimenopausal females. Many exosome proteins have been proposed as diagnostic biomarkers for ovarian cancer, endometriosis, PCOS, and preeclampsia and can be detected in ovarian fluids via highly sensitive detection assays. With further studies, we may be able to ascertain the role of specific follicular fluid exosome proteins in ovarian aging and menopause-related symptoms and determine their diagnostic potential.

## Limitations

This article reviews various studies on the role of follicular fluid exosomes in ovarian function and aging. However, research on their specific changes and functions during menopause is limited. Comparative studies analyzing FF-EV profiles in young and menopausal individuals are essential to clarify their role. Menopause is a highly individual experience, with symptoms varying in severity and type. Additionally, due to lifestyle, genetics, and other ailments, the onset of menopause differs. This makes characterization of exosomes and exosome-related aging mechanisms challenging. The isolation of FF exosomes presents significant challenges. It is a costly and labor-intensive process, and the high protein content in the follicular fluid may interfere with exosome purification. Additionally, the yield and characteristics of exosomes can vary depending on the isolation method and source [28]. As a result, using an increase in exosome numbers with age as a reliable indicator of menopause becomes challenging. Many studies discussed in this review have used animal models, but it is crucial to acknowledge that exosome profiles may differ between animals and humans. Moreover, individual variations in EV profiles arise from differences in physiology and lifestyle. To establish the exosomal proteins discussed in this review as viable biomarkers for menopause, more robust research and clinical validation are needed.

## Data Availability

No datasets were generated or analysed during the current study.

## Abbreviations

FSH: Follicle-stimulating hormone
LH: Luteinizing hormone
AMH: Anti-Mullerian hormone
Wnt: Wingless-related integration site
TGF-β: Transforming growth factor-beta
PI3K: Phosphoinositide 3 kinase
Akt: Protein kinase B
IU/mL: International units per millilitre
pg/mL: Picogram per milliliter
DNA: Deoxyribonucleic acid
RNA: Ribonucleic acid
siRNA: Small interfering ribonucleic acid
miRNA: Microribonucleic acid
mRNA: Messenger ribonucleic acid
β-catenin: Beta-catenin
MAPK: Mitogen activated protein kinase
ERK: Extracellular signal regulated kinase
EV: Extracellular vesicle
FF-EV: Follicular fluid-extracellular vesicles
Exos: Exosomes
FF-exos: Follicular fluid exosomes
CD: Cluster of differentiation
MHC: Major histocompatibility complex
ESCRT: Endosomal sorting complex proteins
ALIX: ALG-interacting protein X
TSG: Tumor susceptibility gene
SNARE: SNAP (soluble N-ethylmaleimide sensitive factor attachment protein) receptor
Rab-GTPases: Ras-associated binding proteinguanosine triphosphatases
G-proteins: Guanine nucleotide-binding proteins
TNF-α: Tumor necrosis factor-alpha
PCOS: Polycystic ovarian syndrome
POI: Premature ovarian insufficiency
POF: Premature ovarian failure
CYP19A1: Aromatase gene
Wntless: Protein wntless homolog or Wis or Evenness Interrupted
BMP: Bone morphogenic protein
GDF: Growth differentiating factor
SMAD: Suppressor of Mothers against Decapentaplegic
R-SMAD: Receptor-regulated SMADs
c-myc: Cellular Myelocytomatosis gene
ROS: Reactive oxygen species
LAP: Latency-associated protein
TSP: Thrombospondin
KLK: Kallikreins
Cx43: Connexin-43
DSL: Delta serrate ligands
Jag: Jagged
Foxl2: Forkhead box L2
lncHOTAIR: Long noncoding HOX transcript antisense RNA
NICD: Intracellular domain of Notch receptors
ARMMs: Arrestin domain-containing protein 1-mediated microvesicles
Kit-L: Stem cell factor
PIP 2: Phosphatidylinositol 4,5-bisphosphate
PIP 3: Phosphatidylinositol (3,4,5)-triphosphate
mTOR: Mechanistic target of rapamycin
FOXO3a: Transcription factor forkhead box O3 protein
BCl: B-cell lymphoma 2 protein
FasL: Fas ligand
PTEN: Phosphate and Tensin Homolog
Ras: Rat sarcoma gene
Raf: Rapidly accelerated fibrosarcoma gene
DDR: DNA damage repair
COL6A1: Collagen alpha 1
HSP: Heat shock protein
ITG: Integrin
IGHM: Immunoglobulin heavy chain constant Mu protein
RAC1: Ras-related C3 botulinum toxin substrate 1
VTN: Vitronectin
YWHAQ: Tyrosine 3-monooxygenase/tryptophan 5-monooxygenase activation protein theta
FGF: Fibroblast growth factor
GH: Growth hormone
IGF: Insulin-like growth factor
GLUT: Glucose transporters
BTG2: B-cell translocation gene 2
DENND1A: DENN domain-containing protein 1A
Hh: Hedgehog
Shh: Sonic hedgehog
Dhh: Desert hedgehog
Ihh: Indian hedgehog
SMO: Smoothened
PTCH: Patched
Gli: Glioma-associated oncogene
cAMP: Cyclic adenosine monophosphate
CREB: cAMP response element binding protein
CRE: cAMP response element
TP53: Tumor protein 53
EGFR: Epithelial growth factor
TSAP6: Tumor suppressor-activated pathway 6
Chmp4C: Charged multivesicular body protein 4C
NLRP3: NOD (Nucleotide oligomerization domain)-like receptor protein 3
NF-κB: Nuclear factor kappa-light-chain enhancer of activated B cells
IL: Interleukin
PKA: Protein kinase A
PKC: Protein kinase C
SIRT: Sirtuin (gene)
Nrf2: Nuclear factor E2-related factor
S100A8: S100 calcium binding protein A8
S100A9: S100 calcium binding protein A9
PRDX: Peroredoxin
CRP: C-reactive protein
KNG: And kininogen
DDT: D‐DOPA‐ chrome decarboxylase
ALDH1A1: Retinal dehydrogenase
KGN: Human ovarian granulosa cell line
RANKL: Receptor activator of NF-κB
NO: Nitric oxide
MMP: Matrix metalloproteinase
